# Wild Relatives of Maize, Rice, Cotton, and Soybean: Treasure Troves for Tolerance to Biotic and Abiotic Stresses

**DOI:** 10.3389/fpls.2018.00886

**Published:** 2018-06-28

**Authors:** Jafar Mammadov, Ramesh Buyyarapu, Satish K. Guttikonda, Kelly Parliament, Ibrokhim Y. Abdurakhmonov, Siva P. Kumpatla

**Affiliations:** ^1^Agriculture Division of DowDuPont™, Corteva Agriscience™, Johnston, IA, United States; ^2^Center of Genomics and Bioinformatics, Academy of Sciences of the Republic of Uzbekistan, Republic of Uzbekistan, Tashkent, Uzbekistan

**Keywords:** crop wild relatives (CWRs), maize, rice, cotton, soybean, tolerance to biotic stress, tolerance to abiotic stress

## Abstract

Global food demand is expected to nearly double by 2050 due to an increase in the world's population. The Green Revolution has played a key role in the past century by increasing agricultural productivity worldwide, however, limited availability and continued depletion of natural resources such as arable land and water will continue to pose a serious challenge for global food security in the coming decades. High yielding varieties with proven tolerance to biotic and abiotic stresses, superior nutritional profiles, and the ability to adapt to the changing environment are needed for continued agricultural sustainability. The narrow genetic base of modern cultivars is becoming a major bottleneck for crop improvement efforts and, therefore, the use of crop wild relatives (CWRs) is a promising approach to enhance genetic diversity of cultivated crops. This article provides a review of the efforts to date on the exploration of CWRs as a source of tolerance to multiple biotic and abiotic stresses in four global crops of importance; maize, rice, cotton, and soybean. In addition to the overview of the repertoire and geographical spread of CWRs in each of the respective crops, we have provided a comprehensive discussion on the morphological and/or genetic basis of the traits along with some examples, when available, of the research in the transfer of traits from CWRs to cultivated varieties. The emergence of modern molecular and genomic technologies has not only accelerated the pace of dissecting the genetics underlying the traits found in CWRs, but also enabled rapid and efficient trait transfer and genome manipulation. The potential and promise of these technologies has also been highlighted in this review.

## Introduction

Understanding the origins of crop plants and their relationships to wild relatives has been a major focus for plant biologists for many years. This knowledge continues to be of great importance in dissecting the process of crop domestication and the ability to leverage wild relatives for crop improvement. Germplasm characterization studies and breeding programs over decades have shown that cultivated plants, in general, have a relatively lower level of tolerance to biotic and abiotic stresses when compared to crop wild relatives (CWRs). One-dimensional selection for increased yield has been hypothesized to result in metabolic resource allocation toward accelerated growth and reproduction and away from plant's tolerance to biotic and abiotic factors (Rosenthal and Dirzo, 1997). Alternatively, plant breeders and population geneticists believe that artificial selection for very small percentage of genes has created breeding bottlenecks that has drastically reduced genetic variation of modern crops and led to the loss of genes derived from CWRs (Hufford et al., 2012). Although tolerance genes have traditionally been considered as negatively correlated with yield (Strauss et al., 2002; Wise, 2007), it has been recently reported that breeding for multiple plant defense traits can be achieved without compromising crop yield (Kaplan et al., 2009).

CWRs offer a diverse array of traits with the potential to decrease the amount of yield loss as a result of biotic and abiotic stresses and pest damage. These CWR- derived resistant traits could be brought into susceptible modern crops through conventional breeding (if there is a sexual compatibility), transgenesis, or other emerging technologies. Introgression of traits of interest from a CWR to a sexually compatible conventional line through traditional breeding could add complications due to the substantial amount of linkage drag resulting from the CWR. However, marker-assisted backcrossing has proven to be a technique capable of rapidly eliminating linkage drag with a minimum number of generations (Peng et al., 2014; Vishwakarma et al., 2014). If biological constraints are creating barriers to transfer desirable loci from CWRs to crops, then transgenesis can be a method of choice. Theoretically, transgenesis is attractive because it eliminates linkage drag, however, practically, it is a very cumbersome and expensive process. Transgenesis also depends on the complexity of the donor genome, availability of an efficient transformation system for the recipient crop, and, last but not least, the final product will have to undergo a lengthy, expensive, and complicated deregulation process.

The use of CWRs in crop improvement has been extensively reviewed by Hajjar and Hodgkin (2007), Prescott-Allen and Prescott-Allen (2013), and Yumurtaci (2015). Hajjar and Hodgkin (2007) and Prescott-Allen and Prescott-Allen (2013) reviews have covered research activities prior to 2005, whereas Yumurtaci (2015) has focused on studies published between 1997 and 2014 covering wheat, barley, maize, and oat. The purpose of this review was to summarize information related to economically important biotic and abiotic traits characterized in CWRs of two monocot crops (maize and rice) and two dicot crops (soybean and cotton).

## Maize

**Figure 1:**
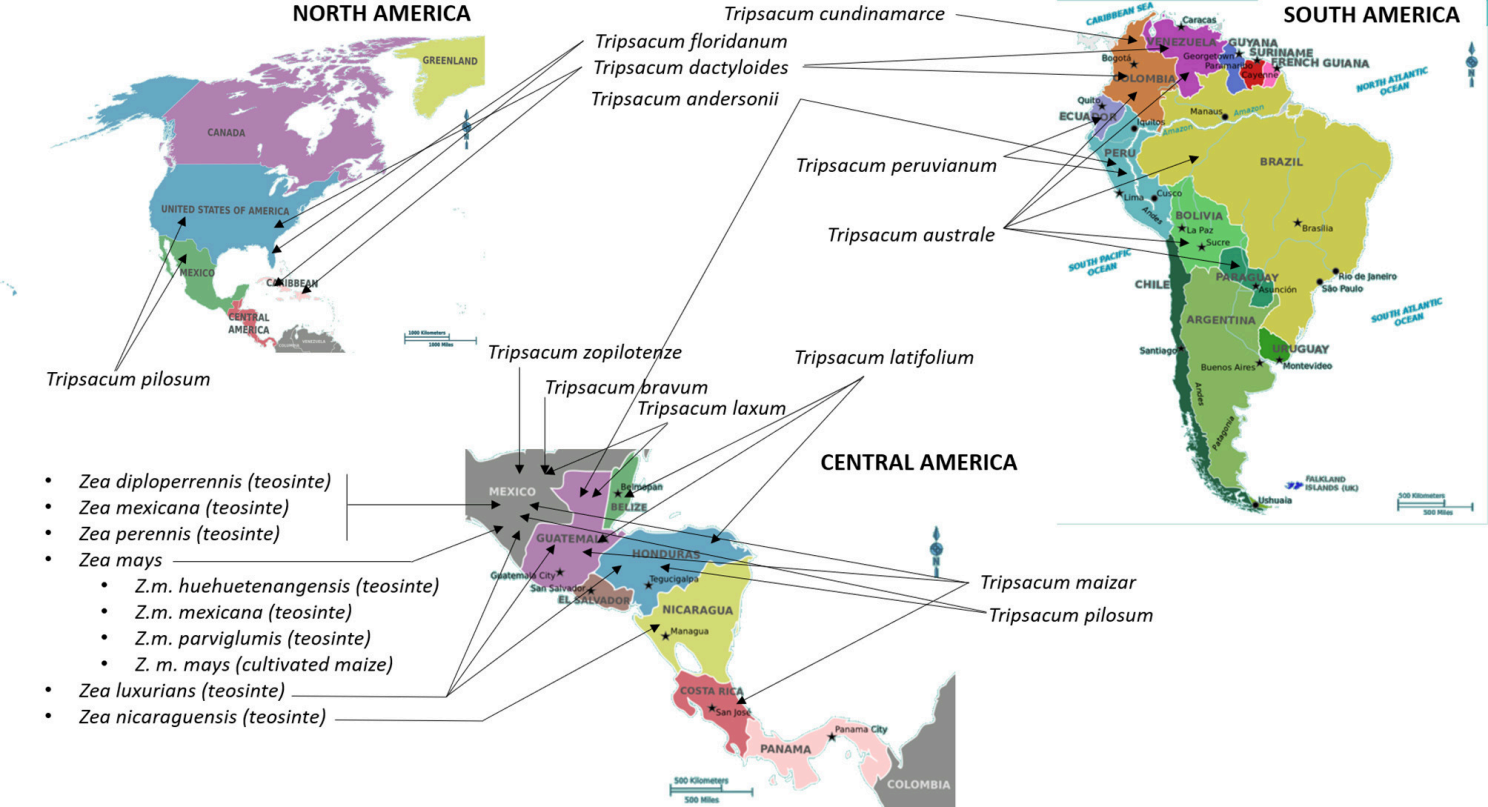
Centers of origin and primary geographical distribution of cultivated maize and its wild relatives. Information on the distribution and centers of origin was gathered from Wilkes (1977), Doebley (1983), Iltis and Benz (2000), and Standley (2015). International Maize and Wheat Improvement Center (CIMMYT) (https://www.cimmyt.org/) is a major maize “living catalog of genetic diversity comprising over 28,000 unique seed collections of maize,” including teosinte species. In addition to CIMMYT, seeds of maize, teosinte, and Tripsacum species are stored and could be obtained from the Germplasm Resources Information Network in Beltsville, USA (https://npgsweb.ars-grin.gov/gringlobal/search.aspx) and N. I. Vavilov Research Institute of Plant Industry in Saint Petersburg, Russia (http://www.vir.nw.ru/).

Maize (*Zea mays* L., ssp. *mays*) is one of the most important crops in the world cultivated primarily for use in animal feed and biofuel. Teosinte (*Z. mays* ssp. *parviglumis* Iltis & Doebley) and *Tripsacum* are two CWRs that have been extensively characterized as donors of economically important traits that could be used for improvement of maize (Table 1). It has taken nearly a century to confirm that Balsas teosinte (*Z. mays* ssp. *parviglumis* Iltis & Doebley) is a progenitor of maize (Matsuoka et al., 2002). Teosinte is a wild grass natively grown in Mexico and some Central American countries including Nicaragua (Iltis and Benz, 2000), Guatemala (Wilkes, 1977), and Honduras (Standley, 2015); refer to Figure 1 for geographical representation. Genus Zea comprises annual species *Zea luxurians*, diploid perennial *Z. diploperennis*, tertraploid perennial *Z. perennis*, and polytypic annual species *Z. mays*, which in turn includes four subspecies; ssp. *mays* (maize), ssp. *mexicana*, ssp. *parviglumis*, and ssp. *huehuetanangensis* (Fukunaga et al., 2005). *Tripsacum* has been considered closely related to *Zea* due to morphological similarities including the highly specialized cupulate fruitcase, and the ability to cross with *Zea* and produce viable but generally infertile hybrids (Galinat, 1961). The genus *Tripsacum* comprises nine species of warm-season, perennial grasses that are native to the area starting in southern Canada (North America) and extending as far south as Chile (South America) (Doebley, 1983; Eubanks, 2006; Figure 1). One species of *Tripsacum* that has been broadly used to generate intergeneric hybrids with maize is *T. dactyloides*, or Eastern gamagrass (De Wet et al., 1981).

**Table 1:** Summary of biotic and abiotic stress tolerance traits of wild relatives of maize (*Zea mays* subsp. *mays*).

**Trait**	**Crop wild relative**	**Putative cause of resistance/tolerance**
**TOLERANCE TO BIOTIC STRESSES**		
**Insect tolerance**		
Tolerance to fall armyworm (*Spodoptera frugiperda*)	*Z. mays* subsp. *parviglumis*	Leaf toughness and leaf trichome
	*Z. diploperennis*	Chemical composition of leaves
	*Z. mays* spp*. parviglumis*	The higher expression of *wip1, RP1*, and *chitinase* genes
	Teosinte (no information related to specific species)	Emission of herbivore-induced volatiles such as indole and a large number of mono- and sesquiterpenes resulted from FAW leaf herbivory attracts larval parasitoids, *Cotesia marginiventris* and *Meteorus laphygmae*
Tolerance to maize spotted stalk borer	*Z. mays* ssp*. mexicana*	Higher concentration of benzoxazinoids (BXs)
	*Z. mays* spp. *mexicana* *Z. mays* spp. *parviglumis* *Z. perennis*	Emission of (*E*)-4,8-Dimethyl-1,3,7-nonatriene resulted from the egg oviposition of the maize spotted stalk borer (*Chilo partellus*) that attracts egg (*Trichogramma bournieri*) and larval (*Cotesia sesamiae*) parasitoids.
Tolerance to western corn rootworm (*Diabrotica v. virgifera)*	Teosinte (no information related to specific species)	Emission of (*E*)-β-caryophyllene by root herbivory that attracts the entomopathogenic nematode *Heterorhabditis megibi*s
	Eastern gamagrass (*Tripsacum dactyloides)*	Unknown
**Disease tolerance**		
Gray leaf spot resistance	*Z. mays* subsp*. parviglumis*	Unknown
Corn smut disease resistance	Teosinte (no information related to specific species)	Unknown
Maize chlorotic dwarf virus resistance	*Z. diploperennis*	Unknown
Maize chlorotic mottle virus resistance	Z. *diploperennis*	Unknown
Maize streak virus resistance	Z. *diploperennis*	Unknown
Maize bushy stunt mycoplasma resistance	Z. *diploperennis*	Unknown
Maize stripe virus resistance	Z. *diploperennis*	Unknown
Maize rayado fino virus resistance	Z. *diploperennis*	Unknown
Northern corn leaf blight resistance	Z. *diploperennis*	Unknown
	Eastern gamagrass	*Ht3* gene
Southern corn leaf blight resistance	Z. *diploperennis*	Unknown
Corn leaf spot diseases resistance	Z. *diploperennis*	Unknown
Rust resistance	Eastern gamagrass	*Rp1*^td^ gene
**Weed tolerance**		
Tolerance to *Striga hermonthica*	*Z. diploperennis*	The production of a signal that inhibits haustoria development on the roots
	Eastern gamagrass	
**TOLERANCE TO ABIOTIC STRESSES**		
Drought tolerance	Eastern gamagrass	Deeply-penetrating root system
Acid soil and aluminum tolerance	Eastern gamagrass	Unknown
Salinity tolerance	Eastern gamagrass	Ability to conserve sodium in the leaves lowering water potential of leaves, maintaining the turgor pressure required for vegetative growth; and lowering the shoot/root rate Mechanism of highly efficient sodium ion release from the tissue
Waterlogging tolerance	*Z. nicaraguensis*	Ability to develop a barrier to radial oxygen loss in basal areas of adventitious roots under stagnant deoxygenated conditions
	*Z. luxurians*	Unknown
	Eastern gamagrass	Constitutive formation of root aerenchyma

### Tolerance to biotic stresses

#### Insect tolerance

The leaves of Balsas teosinte possess certain physical barriers that are believed to make them less desirable for herbivory when compared to cultivated maize. For instance, leaf toughness was reported as a possible reason why fall armyworm (FAW) (*Spodoptera frugiperda*) and leafhopper (*Dalbulus maidis*) prefer cultivated maize for oviposition in order to provide a better food source for freshly hatched larvae (Takahashi et al., 2012; Bellota et al., 2013; Dávila-Flores et al., 2013; Bernal et al., 2015). Another physical barrier against the parasite invasion is the density of the leaf trichome. This is particularly effective in early leaf development where it is reported to play a significant role in preventing damage by FAW in teosinte species as compared to maize lines (Moya-Raygoza, 2016). Pubescence, measured by trichome density, did not significantly decline through domestication transitions as was expected. Research has also demonstrated that trichome density poses very little barrier against *D. maidis* oviposition (Dávila-Flores et al., 2013).

In addition to physical barriers, chemical composition of teosinte leaves in particular, toxic secondary metabolites, could result in tolerance to herbivore insect consumption (Howe and Jander, 2008). It was shown by Farias-Rivera et al. that leaf extracts and residual fiber from *Z. diploperennis* affected the growth of FAW resulting in diminished length and width of pupae and an increased cumulative mortality rate (Farias-Rivera et al., 2003). The increased expression of *wip1, RP1*, and *chitinase* genes is believed to result in a greater tolerance of Balsas teosinte to FAW (Szczepaniec et al., 2013). Teosinte accessions of *Z. mays* ssp*. mexicana* that demonstrated resistance to maize spotted stalk borer (MSSB) produced a higher concentration of benzoxazinoids (BXs) compared to susceptible maize lines. This was hypothesized to be the underlying molecular basis of teosinte resistance to MSSB (Niazi et al., 2014). Benzoxazinoids have also been reported to be involved in resistance of maize to lepidopterans, in particular, European corn borer (Niemeyer, 2009). In contrast, Maag and colleagues rejected the hypothesis that higher concentrations of BXs and maysin make teosinte more insect tolerant than cultivated maize varieties (Maag et al., 2015). Researchers suggested that teosinte tolerance to herbivore insects is more likely controlled by the other defense traits than BXs and maysin.

In maize and teosinte, herbivory induces the emission of volatile compounds (VOCs) that attract beneficial insects which in turn attack pests (McMullen et al., 2009; Lange et al., 2014; Tamiru et al., 2015). The emission of VOCs is not completely a domesticated trait in maize as the crop also uses VOC as a weapon to fight pests such as FAW and leafhoppers by attracting predators and parasitoids (Hoballah et al., 2004). However, teosinte was reported to attract quantitatively more beneficial insects than cultivated maize (Lange et al., 2014). Depending on which organ of maize or teosinte is damaged by herbivory, the spectrum of the beneficial insect attracted changes. Foliage damage releases a blend of VOCs which consists of indole and a large number of mono- and sesquiterpenes, which are strongly expressed after herbivory by Lepidoptera (Köllner et al., 2004). These VOCs attract parasitic wasps such as *Cotesia marginiventris* and *Meteorus laphygmae* which have a tendency to attack FAW (Hoballah et al., 2004). However, the latter parasitic wasp “learned” to recognize these VOCs to detect their source of food (Carroll et al., 2006). Teosinte was also reported to release the VOC (E)-4,8-dimethyl-1,3,7-nonatriene as a response to the spotted stalk borer oviposition that attracted both egg (*Trichogramma bourneiri*) and larval (*Cotesia sesamiae*) parasitoids (Mutyambai et al., 2015). It was also reported that maize and teosinte roots release VOCs in response to herbivory damage by *Coleoptera*. In particular, they release a (*E*)-β-caryophyllene in a response to damage by *D. v. virgifera*, which attracts the entomopathogenic nematode *Heterorhabditis megibi*s (Rasmann et al., 2005). Interestingly, North American maize lines do not emit a (*E*)-β-caryophyllene, whereas European lines do so in response to *D. v. virgifera* attack (Rasmann et al., 2005). Teosinte, particularly *Z. diploperennis*, seems to attract more root damaging insects, including western corn rootworm (WCR) (*Diabrotica virgifera virgifera*) than maize. However, due to larger amount of root biomass in teosinte it is still more tolerant to insect root damage than maize (reviewed by Lange et al., 2014). In contrast to teosinte, eastern gamagrass has been observed to be resistant to WCR and the resistance has been reported to have a quantitative nature (Prischmann et al., 2009). Eastern gamagrass was also identified as a donor of resistance to the maize weevil, *Sitophilus zeamais* Motschulsky, one of the major pests of maize worldwide (Throne and Eubanks, 2015).

#### Disease tolerance

The genetics of resistance of Balsas teosinte to the fungal disease, gray leaf spot (GLS), was studied by Lennon and colleagues using classical quantitative trait loci (QTL) mapping and employing a population of near isogenic lines (Lennon et al., 2016). Using these approaches Lennon et al was able to identify the teosinte-derived GLS resistance QTL located in bin 4.07. Teosinte was also reported to manifest resistance to corn smut disease (Chavan and Smith, 2014). Using genomic *in situ* hybridization technology, Wei et al. (2001) identified the chromosomal locations of the *Z. diploperennis-*derived segments in maize × *Z. diploperennis* crosses that carried loci resistant to fungal diseases including northern corn leaf blight, southern corn leaf blight, and corn leaf spot diseases. In addition, several other species of teosinte such as *Z. mays* ssp*. mexicana* have been shown to be a donor of downy mildew and *Fusarium* resistance (reviewed by Maazou et al., 2017). Perennial teosinte, *Z. diploperennis*, has demonstrated immunity to many viral and micoplasmal diseases, including maize chlorotic dwarf virus, maize chlorotic mottle virus, maize streak virus, maize bushy stunt mycoplasma, maize stripe and rayado fino viruses (Nault and Findley, 1982). Eastern gamagrass can be utilized as a donor of a novel rust resistance gene *Rp1*^td^. This gene was successfully introgressed into a susceptible maize background and proved capable of controlling resistance to rust in the cooler regions of the tropics (Bergquist, 1981). The northern corn leaf blight resistance gene, *Ht3*, is another disease resistance gene that has been successfully introgressed from eastern gamagrass into maize (Hooker, 1981).

#### Weed tolerance

*Striga hermonthica* is very devastating root parasite of maize in Africa. The parasitism of *S. hermonthica* results from the arrays of signal exchanges between the host (maize or sorghum) and the weed. It has been reported that the host plant will produce specific substances, called strigolactones that serve as a seed germination signal for *S. hermonthica* (Awad et al., 2006). In maize, only qualitative resistance to *S. hermonthica* has been reported (Gethi and Smith, 2004; Amusan et al., 2008). Although neither maize nor its wild relatives show 100% resistance to *S. hermonthica, Z. diploperennis* has demonstrated higher level of tolerance to the parasite compared to a majority of maize cultivars (Kling et al., 2000; Yallou et al., 2009). The notable manifestations of *Z. diploperennis'* tolerance to the parasite are (1) resisting the attachment of nearby germinating *S. hermonthica* to its roots, and (2) restricting the penetration of the vascular system of the host by the parasitic seedlings, which would eventually result in host death (Lane et al., 1997; Rich and Ejeta, 2008). Gurney and colleagues demonstrated that eastern gamagrass also possesses tolerance to *S. hermonthica* through the production of a signal that inhibits haustoria development on its roots (Gurney et al., 2003). This finding of resistance of eastern gamagrass to this parasite plant was determined to be quantitatively inherited.

### Tolerance to abiotic stresses

#### Drought tolerance

If a maize plant is subjected to drought stress within a 2–10 week window before anthesis, yield reductions can be substantial because this is a very critical period for ear development (reviewed in Eubanks, 2006). Eastern gamagrass has been known for its ability to tolerate drought stress due to its morphologic and metabolic characteristics. Roots of Tripsacum can penetrate deeply into soil regardless of quality (Clark et al., 1998; Gilker et al., 2002; Gitz et al., 2013). This plant also demonstrates enhanced water use efficiency and photosynthesis during drought stress (Coyne and Bradford, 1985). Although drought tolerance is a complex trait, Eubanks has demonstrated that it can be successfully introgressed into a cultivated maize background (Eubanks, 2006).

#### Acid soil and aluminum tolerance

Soil acidification is a process that occurs naturally due to decomposition of organic matter and air pollution-caused acid rain. Excessive use of nitrogen-containing fertilizers in agriculture can also contribute to an elevated soil acidity. Acidic soil negatively impacts crops by increasing bioavailability of toxic metal ions, including aluminum. Aluminum toxicity in maize is observed at a pH > 5.5 (Von Uexküll and Mutert, 1995) and results in the inhibition of root growth (reviewed in Aggarwal et al., 2015). Eastern gamagrass has been reported as tolerant to aluminum toxicity and efforts to introgress loci controlling tolerance to aluminum toxicity into maize background have been studied (Eubanks, 2006). Although results were promising, no further reports have been published on developing aluminum tolerant maize cultivars. The mechanism of aluminum tolerance in eastern gamagrass remains unknown.

#### Salinity tolerance

Soil salinity negatively affects crops by causing a hyperionic and hyperosmotic stress environment that slows down plant growth and significantly decreases yield (Shrivastava and Kumar, 2015). Eastern gamagrass has shown tolerance to high salt concentrations in soil associated with the ability to conserve sodium in its leaves lowering the water potential and therefore, providing the turgor pressure necessary for vegetative growth (Pesqueira et al., 2003, 2006). Additional benefits of eastern gamagrass include lowering the shoot/root rate which is a favorable aspect for plant water balance. Another study attributed salinity tolerance of eastern gamagrass to the ability of highly efficient sodium ion release from the tissue (Shavrukov and Sokolov, 2015).

#### Waterlogging tolerance

*Z. nicaraguensis* grows in the coastal plains of Nicaragua and is known for higher waterlogging tolerance than any other species of Zea (Iltis and Benz, 2000). Mano and his colleagues identified the genetic components underlying three major traits that makes *Z. nicaraguensis* waterlogging tolerant: (1) formation of constitutive aerenchyma in drained soil; (2) the ability to grow adventitious roots at the soil surface in waterlogged conditions; and (3) tolerance to reduced soil toxins (Mano et al., 2007, 2009; Mano and Omori, 2008). However, in recent research they did not find a significant correlation between the ability to form constitutive aerenchyma and waterlogging tolerance and, therefore, indicated the presence of other flood tolerance-related traits (Mano and Omori, 2013). Recently Abiko and colleagues concluded that *Z. nicaraguensis* has an ability to develop a barrier to radial oxygen loss (ROL) in basal areas of adventitious roots under stagnant deoxygenated conditions (Abiko et al., 2012). ROL drives a higher internal oxygen supply to the root tip allowing it to grow in waterlogged soil. Another species of teosinte, *Z. luxurians*, was declared a source of a putative waterlogging tolerance due to the nodal root angle (Omori and Mano, 2007). Eastern gamagrass exhibits waterlogging tolerance due to the constitutive formation of root aerenchyma (Hardin, 1994; Comis, 1997; Ray et al., 1998), the tissue that allows oxygen to penetrate to the distal regions of the roots (Mujeeb-Kazi and Jewell, 1985; Gitz et al., 2013). Attempts to introgress waterlogging tolerance into maize through conventional breeding have not been successful due to the complexity of the trait inheritance (Ray et al., 1999).

## Rice

Rice (*Oryza sativa* L.) is the world's most important staple food crop contributing to the majority of dietary intake for nearly half of the global population. Biotic and abiotic stresses have seriously affected rice production in recent times due to changing climatic conditions and non-durability of resistance incorporated into cultivars (Normile, 2008). Genetic variability for resistance/tolerance to these stresses is limited in the germplasm of cultivated rice, however, wild species of *Oryza* are considered rich sources of unexplored genes for these traits (Jena, 2010; Sanchez et al., 2013; Shakiba and Eizenga, 2014). Identification and transfer of these genes from wild species to modern cultivars is, therefore, an attractive option.

**Figure 2:**
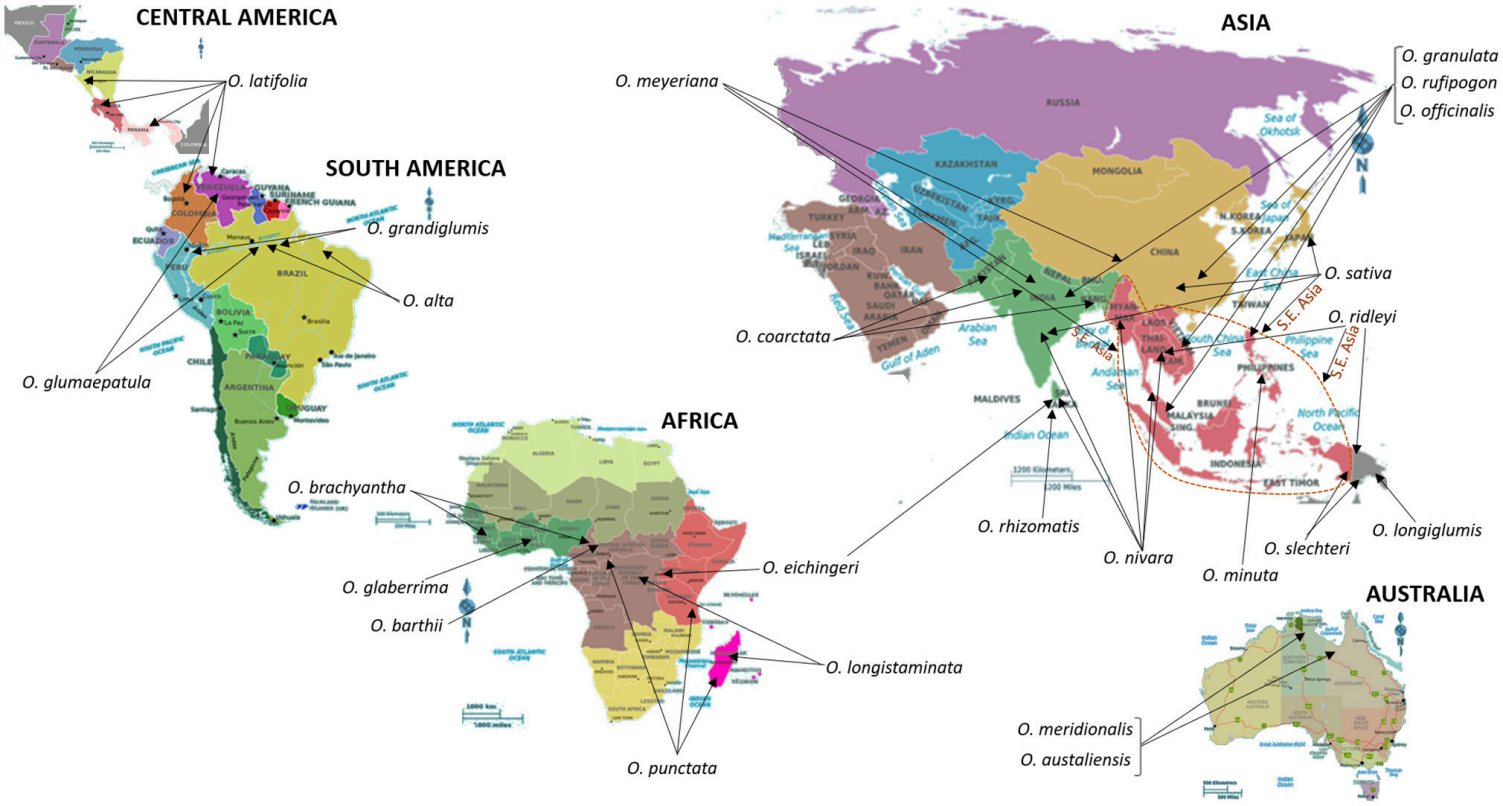
Centers of origin and/or primary sites of diversity and distribution of wild relatives of Rice. South East Asia (S.E. Asia), a key region for the origin and distribution of several wild relatives of Rice, is indicated by a dotted line. Information on the distribution and centers of origin was gathered from Shakiba and Eizenga (2014), and references therein. “The International Rice Genebank, maintained by International Rice Research Institute, holds more than 124,000 rice accessions that include modern and traditional varieties and wild relatives of rice. It is the biggest collection of rice genetic diversity in the world. Countries from all over the world have sent their rice samples to IRRI for safe keeping as well as for sharing” (http://irri.org/). In addition to IRRI, the Germplasm Resources Information Network in Beltsville, USA (https://npgsweb.ars-grin.gov/gringlobal/search.aspx) also stores and distributes genetic material of cultivated rice and its wild relatives.

The genus *Oryza* consists of 24 species of which two, *Oryza sativa* L. and *Oryza glaberrima* S., are cultivated and the remaining are wild relatives distributed around the world (Sanchez et al., 2013; Shakiba and Eizenga, 2014); refer to Figure 2 for geographical resprentations of these locations. Based on taxonomy, isozyme, and DNA marker analysis, and characterization of genome sequences, the species of the genus *Oryza* were assembled into several groups. These groups include *O. sativa, O. ridleyi, O. granulata*, and *O. officinalis*. The species belonging to the *O. sativa* group include the cultivated species, *O. sativa* L. and *O. glaberrima* S., which are diploids and possess the AA genome type (2n = 24). Species belonging to groups farther away from *O. sativa* are diploids or tetraploids and contain other genome types (Sanchez et al., 2013; Shakiba and Eizenga, 2014).

Early breeding efforts for introgressing genes of interest from wild relatives into cultivated species of rice were intentionally focused on wild species with the AA genome because F1 hybrids derived from these crosses display regular chromosome pairing and recombination. In contrast, hybrids between cultivated and wild species that possess alternative genomes (for example, AA genome species crossed with non-AA genome species) are typically more difficult to generate due to incompatibility issues resulting in sub-optimal crossability and non-viable embryos (Brar and Khush, 1997; McCouch et al., 2007). However, embryo rescue can be used to produce male-sterile interspecific F1 hybrids for utilization in a backcrossing program resulting in fertile diploid plants (Jena, 2010). Several tools can be used to enable chromosomal assignment, dissection of traits in wild species, and gene transfer from wild to cultivated species including: monosomic alien addition lines, disomic introgression lines, chromosome segment substitution lines, and backcross inbred lines (Ali et al., 2010; Jena, 2010).

### Tolerance to biotic stresses

#### Insect tolerance

The brown planthopper (BPH), *Nilaparvata lugens* Stål, is a migratory insect that has become the most devastating pest of rice. In addition to causing severe plant damage resulting in significant production losses, BPH also transmits two disease causing viruses, rice grassy stunt virus and rice ragged stunt virus. Although control of BPH using pesticides such as imidacloprid is effective, host-plant resistance is the preferred method. Host-plant resistance has additional benefits including reducing production costs and the risk of possible environmental contamination when compared to chemical control of BPH (Tanaka et al., 2000; Hu et al., 2016).

Several studies indicate that wild species are an important source of planthopper resistance genes (Jena, 2010; Fujita et al., 2013; Hu et al., 2016; Sarao et al., 2016) (Table 2). Studies on the exploration of resistance genes to date have identified a total of 30 *Bph* resistance genes from *indica* and wild species of rice (Fujita et al., 2013; Hu et al., 2016; Ling and Weilin, 2016). Out of the 30 *Bph* resistance genes more than a dozen are from wild relatives. As of today, four resistance genes have been cloned including, *Bph14* and *bph29*, which are from *O. officinalis* and *O. rufipogon*, respectively. Parallel to gene identification and cloning efforts, introgressions of BPH resistance into cultivated/elite lines has also been performed (Table 2; Ram et al., 2010; Sarao et al., 2016).

**Table 2:** Summary of biotic and abiotic stress tolerance traits of wild relatives of rice (*Oryza sativa*).

**Traits**	**Crop wild relative and respective genomes**	**Resistance/tolerance genes, gene loci, and QTL identified in wild species**
**TOLERANCE TO BIOTIC STRESSES**		
**Insect tolerance**		
Brown planthopper resistance	*O. nivara* (AA) *O. punctata* (BB/BBCC) *O. longistaminata* (AA) *O. barthii* (AA) *O. rufipogon* (AA) *O. officinalis* (CC) *O. austaliensis* (EE) *O. minuta* (BBCC) *O. latifolia* (CCDD) *O. glaberimma* (AA)	*Bph10* and *Bph18(t)* (*O. australiensis*); *bph11(t), bph12(t), Bph13(t), Bph14*, and *Bph15* (*O. officinalis*); *Bph12* (*O. latifolia*); *Bph16(t), Bph20(t), Bph21(t)*, and *Bph23(t)* (*O. minuta*); *Bph22(t)* (*O. glaberrima*); *Bph24(t), bph29* and *Bph30* (*O. rufipogon*)
**Disease tolerance**		
Blast resistance	*O. minuta* (BBCC) *O. autraliensis* (EE) *O. rufipogon* (AA) *O. rhizomatis(CC)*	~100 resistance (R) genes and 350+ QTL; Three major R gene clusters (*Piz, Pik*, and *Pita*) were subjected to extensive characterization
Bacterial blight resistance	*O. longistaminata* (AA) *O. rufipogon* (AA) *O. minuta* (BBCC) *O. officinalis* (CC) *O. nivara* (AA) *O. brachyantha* (FF)	~41 resistance genes have been reported; *Xa21* (*O. longistaminata*); *Xa23* (*O. rufipogon*); *Xa27* (*O. minuta*); *Xa29(t)* (*O. officinalis*); *Xa30(t), Xa38* (*O. nivara*); *Xa34(t)* (*O. brachyantha*)
Rice grassy stunt virus resistance	*O. nivara* (AA)	Gs (*O. nivara*)
Rice tungro bacilliform virus tolerance	*O. longistaminata* (AA) *O. rufipogon* (AA)	Ongoing efforts include gene/QTL identification and using *O. longistaminata* and *O. rufipogon* as donors in developing tolerant lines
**TOLERANCE TO ABIOTIC STRESSES**		
Drought and heat tolerance	*O. glaberrima* (AA) *O. barthii* (AA) *O. meridionalis* (AA) *O. australiensis* (EE) *O. longistaminata (AA)*	Ongoing efforts include gene/QTL identification and using donors such as *O. meridionalis* in developing tolerant lines
Acid soil and aluminum tolerance	*O. rufipogon* (AA)	Several QTL identified in *O. rufipogon*
Salinity tolerance	*Porteresia coarctata* (*O.coarctata*) (KKLL)	*Porteresia coarctata* being subjected to genomic/ transcriptomic analysis to identify key genes/pathways
Cold tolerance	*O. rufipogon* (AA)	QTL identified in *O. rufipogon*

#### Disease tolerance

Rice blast is considered the most serious and economically important disease caused by a fungal pathogen in rice crop. The causative agent is *Magnaporthe oryzae (M. oryzae)*. Although *M. oryzae* infects other grasses, the primary host is rice. Since the late nineteenth century when rice blast was first observed in the United States it has been reported in 85 rice-growing countries around the globe and caused severe economic damage in several of these countries (Wang et al., 2014). Since host plant resistance is the preferred mode of control efforts in rice blast research has focused on identifying resistance genes and QTLs in wild species. To date, more than 100 resistance genes (called *R* genes) and over 350 QTL regions have been identified (Wang et al., 2014; Ashkani et al., 2015; Vasudevan et al., 2015). Out of all of the *R* genes identified, 96% were found in *japonica* and *indica* cultivars, and the remaining 4% in wild relatives (Wang et al., 2014). Studies on *R* gene mapping revealed their presence in all rice chromosomes except chromosome 3. Three major *R* gene clusters, *Piz, Pik*, and *Pita*, were identified and mapped to chromosomes 6, 11, and 12, respectively. These clusters have been the focus of extensive characterization research over the years and apart from a few exceptions, most *R* genes cloned have been found to encode nucleotide binding site-leucine-rich repeat (NBS-LRR) proteins, similar to other plant *R* genes known to date (Wang et al., 2014). Wild species including, *O. minuta, O. autraliensis, O. rufipogon*, and *O. rhizomatis*, have been found to harbor resistance genes to rice blast. These genes have been introgressed into susceptible lines and proven effective against rice blast disease (Table 2; Sharma et al., 2012; Wang et al., 2014; Ashkani et al., 2015). Although many varieties were developed containing a single resistance gene resistance it was broken down in 3–5 years of release due to the emergence of new races of rice blast. Therefore, it is important to develop varieties containing broad spectrum and/or durable by stacking different *R* genes with overlapping resistance spectra (Sharma et al., 2012). This requires the availability of a pool of *R* genes to utilize. Since a very small portion (4%) of the *R* genes identified to date are from wild species, exploring them further is important.

Bacterial blight, caused by the pathogen *Xanthomonas oryzae* pv. *oryzae (Xoo)*, is endemic to most of the rice growing regions in Asia and West Africa and has recorded yield losses as high as 75% in India, Indonesia, and the Philippines (Shakiba and Eizenga, 2014). Extensive research efforts to date have resulted in the identification of 41 reported resistance genes, of which eight have been characterized (Ellur et al., 2016). Table 2 lists some of the wild relatives and the blight resistance genes identified in their genomes. A widely used resistance gene, *Xa21*, was isolated using a positional cloning strategy, and found to encode a receptor kinase-like protein (Song et al., 1995). It was one of the first genes tagged with DNA markers and transferred to many rice cultivars using marker-assisted breeding, however, in China, new strains of *X. oryzae* pv. *oryzae* broke the resistance conferred by *Xa21* highlighting the need to explore more novel genes with durable resistance. Out of all known resistance genes *Xa23*, a single, completely dominant gene identified from *O. rufipogon*, has recorded efficacy at all growth stages of rice, possesses broad spectrum resistance, and was acknowledged as the most promising gene highly resistant to all the 20 strains of *Xoo* (Zhang et al., 2001; Zhang and Xie, 2014). A novel resistance gene, *Xa38*, has been identified recently in *O. nivara* and its deployment in varieties is expected to counter the breakdown of resistance to *Xoo* (Ellur et al., 2016).

More than 20 viruses infect rice and a majority of them use insects as vectors for their transmission. Two virus-associated diseases that cause significant damage to rice are: rice grassy stunt disease; caused by rice grassy stunt virus (RGSV), and rice tungro disease; caused by mixed infection of rice tungro bacilliform virus (RTBV) and rice tungro spherical virus (RTSV). RGSV uses rice brown planthopper as its vector for transmission. Infection results in stunted plants that produce very few to no panicles with deformed grains. Screening of thousands of accessions of cultivated and wild species led to the identification of *O. nivara* (AA-genome) as the only source of resistance due to a single dominant gene, *Gs*. The transfer of RGSV resistance from *O. nivara* into cultivated *O. sativa* represented the first successful introduction of a useful, agronomic gene from a wild to cultivated species of rice (Khush et al., 1977). Rice tungro disease is another economically important viral disease in Southern and Southeastern Asia and is transmitted by green leafhopper *Nephotettix virescens*. Of the two Rice tungro disease-causing viruses, RTBV and RTSV, RTBV is the main cause of symptoms. Cultivated rice germplasm has limited variability for resistance to RTBV. Tolerance to RTBV has been identified in two wild species, *O. longistaminata* and *O. rufipogon*, and many tungro-tolerant lines have been developed utilizing them as donors (Khush et al., 2004; Table 2).

### Tolerance to abiotic stresses

#### Drought and heat tolerance

Drought is one of the main environmental stressors that reduces agricultural productivity in rice. Exploratory studies have been performed to better understand the effects of drought using backcross inbred lines from a cross between an *O. sativa* line, WAB56-104, and an *O. glaberrima* line, CG14 (Ndjiondjop et al., 2010). The findings indicated that drought reduces grain yield and adversely affects yield stability by affecting several morphological traits, including delay of flowering time and plant maturity. Although several QTLs for drought tolerance have been identified in *O. sativa* and the underlying genes cloned, wild relatives are considered to harbor stronger/novel alternatives and, therefore, several promising species are being investigated (Ndjiondjop et al., 2010; Menguer et al., 2017; Table 2). For example, although low yielding, *O. glaberrima*, has been found to be an excellent source of tolerance for drought. Screening several accessions of three wild species, *O. barthii, O. australiensis*, and *O. meridionalis*, has shown variability for two key traits; plant height and tillering ability. These species, therefore, can be used as donors in breeding programs aimed at developing tolerance to drought and heat (Ndjiondjop et al., 2010; Sanchez et al., 2013). While the exploration of candidate wild relatives continues, some donors have already been used in crossing programs to develop varieties tolerant to heat (Sanchez et al., 2013).

#### Acid soil and aluminum tolerance

Aluminum toxicity is of utmost concern when rice is grown in acidic soils since it adversely effects root development, water and nutrient uptake, and growth resulting yield loss. Tolerance to aluminum is a quantitative trait with high variability among various rice species and therefore, the identification and introgression of an associated QTL into cultivated varieties is a very promising option. IRGC106424, an accession of *O. rufipogon*, was originally identified growing in acidic soils in Vietnam and has since proven to be a valuable resource for imparting aluminum tolerance to rice cultivars (Sanchez et al., 2013; Table 2). The evaluation of recombinant inbred lines derived from *indica* and *O. rufipogon* crosses by Nguyen et al. led to the identification of several QTLs for stress-associated root length, including a major QTL on chromosome 3 (Nguyen et al., 2003). An acid sulfate tolerant rice variety, AS996, has been developed by introgressing tolerance from *O. rufipogon* into the IR64 background (Sanchez et al., 2013). Studies on understanding the physiological and genetic basis of aluminum tolerance in *O. rufipogon* have indicated an association with cell division and altered photosynthesis (Cao et al., 2011).

#### Salinity tolerance

While high soil salinity is a serious problem for most of the major agricultural crops, it is especially important for rice which is one of the most salt-sensitive crops. High salinity is reported to reduce seed germination, decrease growth, and survival of seedlings, damage chloroplast structure, reduce photosynthesis and negatively impact seed set and grain yield. *Porteresia coarctata* (*Oryza coarctata*), an Asian halophyte and wild relative of rice, occurring in coastal environments shows high salinity and submergence tolerance (Zhang and Xie, 2014; Table 2). Transcriptome sequence of *P. coarctata* suggested salinity and submergence tolerance in this species is due to substantial transcriptional reprogramming (Garg et al., 2014). Such efforts pave the way for dissecting the mechanism(s) of tolerance and identifying key genes for salinity tolerance in rice. Ultimately, resistance could be transferred to *O. sativa* through bridge crossing or leveraging molecular techniques.

#### Cold tolerance

Cold tolerance is a concern for rice cultivation and productivity since stress induced by low temperatures can adversely affect germination, growth, and pollen development (Andaya and Tai, 2007). Initial efforts focused on identifying variability for cold tolerance in *indica* and *japonica* subspecies, which are better adapted to tropical and temperate climates. Large scale screening of *indica* and *japonica* cultivars demonstrated that *japonica* rice possesses relatively high tolerance to low temperatures at seedling, vegetative and reproductive stages and, therefore, efforts focused on identification of underlying QTL(s) on progeny derived from *japonica* and *indica* crosses (Lou et al., 2007; Zeng et al., 2009; Cruz et al., 2013). In addition, the wild relative *O. rufipogon* was also found to harbor a QTL for cold tolerance and, therefore, an ideal donor for breeding programs (Koseki et al., 2010; Table 2). QTL analysis of progeny from crosses between *O. rufipogon* (cold tolerant) and *indica* (cold sensitive) or *japonica* cultivars yielded a major QTL explaining 40% of the phenotypic variation (Koseki et al., 2010). This identified cold tolerant QTL was later fine mapped.

## Cotton

**Figure 3:**
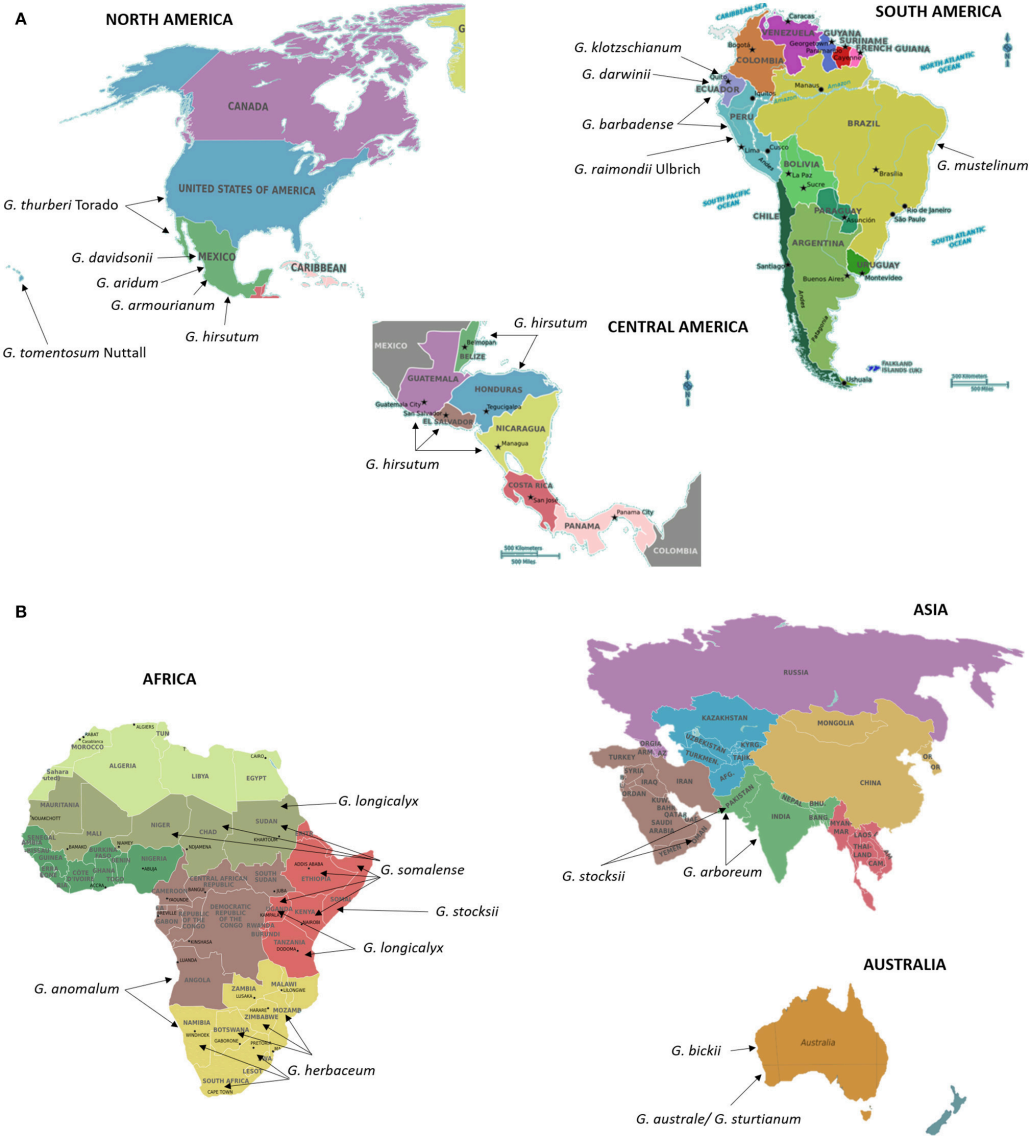
**(AB)** Centers of origin or primary geographic distribution sites of cultivated cotton species *G. hirsutum* L. **(A)**, *G. barbadense* L **(A)**, *G. arboreum* L. **(B)**, and *G. herbaceum* L. **(B)**, and their wild relatives **(A,B)**. Information on the distribution and centers of origin was gathered from Fryxell (1992) and Wendel and Cronn (2003). Seeds of cotton and its wild relatives are stored and could be obtained from the Germplasm Resources Information Network in Beltsville, USA (https://npgsweb.ars-grin.gov/gringlobal/search.aspx).

Cotton (*Gossypium* spp.) is the most commonly grown natural fiber and oil seed crop throughout the world. Understanding the evolutionary history of cotton is as equally important as that of developing new cultivars. The cotton genus (*Gossypium*) includes ~50 species, including five allotetraploids, and other diploid species distributed across Africa, Australia, Central and South America, the Galapagos Islands, Hawaii, the Indian sub-continent, and Arabia (Fryxell, 1992; Wendel and Cronn, 2003). Figures 3A,B show the native locations of these cotton species across the world. *Gossypium* species express wide morphological differences from herbivorous perennials to small trees with enormous diversity in vegetative and reproductive features (Wendel and Cronn, 2003). Most of the world's textile fiber is derived from four species of the genus *Gossypium*, including *two* New World tetraploid species, *G. hirsutum* L. and *G. barbadense* L (Figure 3A) and two Old World Asian-African diploids *G. arboreum* L., *G. herbaceum* L. (Figure 3B). Diploid *Gossypium* species diverged into multiple genomes designated A–G, and K, based on meiotic chromosomal pairing behavior (Wendel and Cronn, 2003). The new world tetraploid species arose ~1–2 million years ago resulting from hybridization of A-genome diploid species, *G. herbaceum* and *G. arboreum* (2n = 2x = 26), with “D-genome” diploid species, *G. raimondii* Ulbrich and *G. gossipioides* L. (2n = 2x = 26) (Wendel, 1989; Wendel and Cronn, 2003). Currently, a diverse *Gossypium* germplasm containing wild species, race and seed stocks is conserved through national germplasm banks worldwide. The inventory of the World Cotton Germplasm Resources shows there are ~53,000–63,946 world cotton accessions preserved in major cotton-growing countries (Campbell et al., 2010; Abdurakhmonov, 2014, 2016). The wealth of the diversity available among wild and cultivated cotton species is a valuable resource for cotton breeders to hasten selection of traits of agronomic importance. Although the transfer of beneficial traits from wild diploid species into cultivated tetraploid cottons is a challenging task, many researchers have been successful through the development of interspecific hybrids involving donor and recipient genotype. These interspecific hybrids have been developed through bridge crosses or other means, including finding genetic recombinants between donor and recipient genotype chromosomes using cytogenetics and other techniques (Robinson et al., 2004a; Konan et al., 2007; Table 3).

**Table 3:** Summary of biotic and abiotic stress tolerance traits of wild relatives of cultivated cotton species.

**Trait**	**Crop wild relative**	**Putative cause of resistance/tolerance**
**TOLERANCE TO BIOTIC STRESSES**		
**Insect tolerance**		
*Helicoverpas* Spp.	*G. somalense*	Smooth leaf type
Tolerance to Jassids	*G. barbadense* “Carpulla” *G. barbadense* “Tanguis” *G. hirsutum* “MU 8*b”* *G. hirsutum* var. “Marie-galante” *G. hirsutum* var. “St. Ignatius” *G. raimondii*	Hairiness trait controlled by genes, designated as *H*_1_(“Carpulla,” “Tanguis,” “MU 8b”,” “Marie-galante,” and “St. Ignatius”) and *H*_6_ (*G. raimondii*) Low Tannin/High Phenol content (*G. anomalum, G. armourianum, G. raimondii, G. davidsoni, G. thurberi*)
Tolerance to fleahopper	*G. hirsutum* “Pilose”	Pilose trait and square structure impacting stylet penetration
Tolerance to thrips	*G. tomentosum* *G. barbadense* “Pima S-7”	Unknown
Nectariless	*G. sturtianum*	Cotton plant without the extrafloral and floral nectary glands do not attract insects
Glandless-seed and glanded-plant	*G. tomentosum*	The presence of pigment glands filled with gossypol and its derivatives helps to protect cotton plants from phytophagous pests
**Nematode tolerance**		
Reniform nematode resistance	*G. longicalyx* *G. somalense* *G. stocksii* *G. arboretum* *G. barbadense* “GB713” cultivar	Resistance gene in Chr 11 from *G. longicalyx* and in 21 from *G. barbadense* “GB713”
Root-knot nematode resistance	*G. hirsutum* “M-315 RNR”	Resistance genes in Chr-11 and Chr 14
**Disease tolerance**		
Bacterial blight resistance	*G. arboreum*	Bacterial blight resistance gene, *B6*.
Rust resistance	*G. anomalum*	
Cotton Leaf Curl Virus resistance	*G. stocksii* *G. herbaceum*	Unknown
Fusarium Wilt resistance	*G. austral* *G. sturtianum* *G. darwinii*	Unknown
Verticillium Wilt resistance	*G. austral* *G. thurberi* *G. darwinii*	Unknown
**TOLERANCE TO ABIOTIC STRESSES**		
Drought tolerance	*G. tomentosum* *G. herbaceum* *G. darwinii*	Unknown
Salt tolerance	*G. tomentosum* *G. davidsonii* *G. aridum*	Unknown
Heat tolerance	*G. tomentosum*	Unknown

### Tolerance to biotic stresses

#### Insect tolerance

Cotton attracts many pests and host tolerance can be improved through morphological and biochemical traits including hairiness, okra leaf shape, nectariless, and polyphenol compounds which may confer broad-spectrum insect tolerance. Cotton displays various densities of trichomes (pubescence), on leaves and stem. Depending on the density of trichomes, pubescence phenotypes are rated as smooth (no trichomes), hirsute (moderate density), and pilose (high density) and most of the modern cotton cultivars belong to smooth category (Wright et al., 1999). Though the smooth leaf trait is related to reduced oviposition by the *Heliothis* spp. (Hassan et al., 1990), and is a desirable feature to minimize the heavy reliance on pesticide usage, it may increase susceptibility to other pests such as jassids. However, several explorations of jassid resistance have demonstrated that hairs of reasonable length and density presented on the underside of a cotton leaf can impart immunity to jassids. A basic, partially dominant, hairiness gene, designated *H*_1_, is shown to be responsible for the resistance of two *G. barbadense* perennial types, Carpulla and Tanguis, a commercial type from India, MU 8*b* (*G. hirsutum*), and the “St Ignatius” variety (*G. hirsutum* var. *marie-galante*) from British Guiana (Zafar et al., 2009). Another hairiness contributing gene (*H*_6_) was successfully transferred from *G. raimondii* to the *G. hirsutum* race *punctatum* (Saunders, 1965). Cotton plants with high phenolic content showed low incidence of jassids. Diploid species such as *G. anomalum* (B_1_), *G. armourianum* (D_2_), *G. raimondii* (D_5_), *G. davidsoni* (D_3_), and *G. thurberi* (D_1_) with low tannin and high phenol contents are genotypes less susceptible to jassids (Shinde et al., 2014).

Cotton fleahopper, *Pseudatomoscelis seriatus*, is a piercing–sucking pest of cotton that feeds preferentially on developing flower buds, called squares. It is observed that square structure along with the reproductive tissue morphology affect the stylet penetration of fleahoppers and thus contribute to resistance in cotton genotypes derived from the crosses between fleahopper resistant “Pilose” and other and high-yielding susceptible lines (McLoud et al., 2016).

Thrips are one of the most damaging early growing season insects and can cause yield losses up to 1% in spite of one insecticide application. Among the five allotetraploid cotton species, *G. tomentosum* with the Pilose trait was the most resistant, followed by *G. mustelinum, G. barbadense*, and *G. darwinii*, and *G. hirsutum* was the most susceptible (Zhang et al., 2013). Zhang et al. (2013) were able to transfer thrips resistance from Pima S-7, a *G. barbadense* accession, into Upland cotton which is ideal because host resistance is the most attractive strategy for the control of thrip damage.

Upland cotton has leaf, extrafloral, and floral nectary glands which secrete nectar that attracts many insects. In contrast, *G. tomentosum* Nuttall, a wild cotton tetraploid species native to Hawaii, does not contain leaf or extrafloral nectaries. Through interspecific crosses between *G. hirsutum* and *G. tomentosum*, the absence of leaf and extrafloral nectaries (nectariless trait) was transferred to Upland cotton (Meyer and Meyer, 1961). The presence of a plant alkaloid, gossypol, and its derivatives above 0.02–0.04% (WHO/FAO recommended limits) in cotton seed oil and meal limits its usage as food and feed. However, gossypol and its derivatives in pigment glands protect cotton plants from phytophagous insects. Thus, a desirable cotton plant would have glandless seeds on a glanded plant to protect cotton plants from phytophagous pests, as well as, increase the value of the seed derived products (Zhu et al., 2005). Two tri-specific hybrids were created using either *G. thurberi* Torado (2*n* = 2*x* = 26, D_1_ genome) or *G. raimondii* Ulbrich (2*n* = 2*x* = 26, D_5_ genome) as bridge species to introgress the “glandless-seed and glanded-plant” trait from *G. sturtianum* Willis (2*n* = 2*x* = 26, C_1_ genome) into the upland cotton *G. hirsutum* L. Further crosses of these tri-specific hybrids by *G. hirsutum* produced the first backcross progenies (BC_l_) to serve as donors of these traits for other elite cotton lines (Bi et al., 1998). A similar introgression effort was attempted from *G. bickii* into upland cotton by crossing the amphidiploid F_1_ (*G. arboreum* × *G. bickii*) as female with various glanded accessions of *G. hirsutum* as male parents (Zhu et al., 2005).

#### Nematode tolerance

In recent years, reniform nematode (*Rotylenchulus reniformis* Linford and Oliveira), has been causing significant economic damage to cotton industry with losses exceeding $100 M annually (Blasingame, 2005). Germplasm mining into the collection of 2,000 primitive *G. hirsutum* accessions for sources of reniform nematode resistance (Robinson and Percival, 1997) resulted in the identification of only six accessions with moderate resistance and highly resistant cultivars were non-existent (Robinson et al., 2004b). Even though highly resistant sources were previously identified in wild diploid species, including *G. longicalyx* J.B. Hutch. & B.J.S. Lee; *G. somalense* (Gürke) J.B. Hutch.; *G. stocksii* Mast.; *G. arboreum* L.; and tetraploid species of *G. barbadense* L. (Yik and Birchfield, 1984), genetic incompatibility, ploidy, climbing growth habit, photoperiodism, and agronomic issues were major challenges for introgressing the resistance trait from these alien genomes. Through the development of three-species hybrids named HLA [(*G. hirsutum* × *G. longicalyx*)^2^ × *G. armourianum*] and HHL [(*G. hirsutum* × *G. herbaceum*)^2^ × *G. longicalyx*] at USDA-ARS, Texas in the United States (Robinson et al., 2004a) and HTL [(*G. hirsutum* × *G. thurberi*)^2^ × *G. longicalyx*] at Gembloux Agricultural University, Belgium (Konan et al., 2007), researchers were successful in identifying donor plants which were fertile and had reniform nematode resistance. In another effort to simultaneously provide host tolerance for two major nematode pests in cotton (reniform and root-knot nematodes), Bell et al. (2015), were successful in introgressing reniform nematode resistance from GB 713, a photoperiodic *G. barbadense* race stock accession, into M-315 RNR, a root-knot nematode resistance accession. Lonren-1, Lonren-2, and Barbren were the agronomically improved Upland cotton germplasm releases by USDA-ARS, College Station, that have reniform nematode resistance derived from *G. longicalyx* (Bell et al., 2014) and *G. barbadense* (Bell et al., 2015), respectively. Such resistant sources add significant value for breeding programs to improve their germplasm.

#### Disease tolerance

Bacterial blight, caused by *Xanthomonas axonopodis* pv. *malvacearum*, is a key disease in many parts of the world. *Gossypium* taxa harboring A-genome were shown to possess near-immunity for this pathogen and, therefore, these sources are valuable for introgression into cultivated tetraploids. Bacterial blight resistance gene, *B6*, found in *G. arboreum* was successfully transferred into *G. barbadense* (Zafar et al., 2009). Cotton rust resistance genes were successfully transferred from *G. anomalum* into *G. hirsutum* through interspecific hybridization, polyploidy induction and continuous screening for resistance in back-cross populations (Blank and Leathers, 1963).

Cotton leaf curl virus (CLCuV) is the wide spread and most damaging disease in northern India and Pakistan and is capable of causing yield loss up to 90%. Since none of the existing *G. hirsutum* varieties have recorded resistance to CLCuV, wild relatives have been explored. *G. arboreum*, was used as a donor of CLCuV resistance to transfer it to *G. hirsutum* using conventional hybridization and backcrossing. In another effort, resistance to CLCuV from *G. stocksii* was successfully introgressed into “MNH-786,” a *G. hirsutum* cultivar, through interspecific hybridization (Nazeer et al., 2014).

*G. australe*, a wild G-genome species possesses resistance to Fusarium wilt and Verticillium wilt diseases apart from other economically important traits such as resistance to aphids and mites. *G. herbaceum*, a cultivated diploid species (A-genome), has resistance to leaf curl virus along with other favorable traits including tolerance to sucking pests and drought tolerance (Liu Q. et al., 2015). Recently, a novel synthetic allotetraploid (A_1_A_1_G_1_G_1_) was developed from two diploid species and capable to lay the foundation for transferring favorable alleles into Upland cotton (Liu Q. et al., 2015). It was reported that throughout Australia no *Fusarium* spores were isolated from *G. sturtianum* stems suggesting that this species might possess Fusarium resistance (Wang et al., 2004), whereas *G. bickii* showed higher affinity with the pathogen (McFadden et al., 2004). *G. thurberi* is another wild species containing high Verticillium wilt tolerance which was supported by research using two-dimensional electrophoresis (2-DE) and tandem time-of-flight mass spectrometry (MALDI-TOF-MS) to identify 57 different proteins in *G. thurberi* infested with *Verticillium dahliae* (Zhao et al., 2012).

### Tolerance to abiotic stresses

*G. tomentosum*, a wild cotton species, is host to many unique agronomic traits, including drought tolerance, salt tolerance, heat tolerance, nectarilessness, insect-pest resistance and lint color. *G. darwinii*, another wild allotetraploid species, with AD_5_ genome has many useful traits, including drought tolerance, fiber fineness, Fusarium wilt, and Verticillium wilt resistance (Liu F. et al., 2015). Soil salinity adversely affects crop growth along with cotton yield and fiber quality. Salt tolerance can improve plant emergency and ensure adequate uniformity stand in cotton. Through an interspecific cross between *G. tomentosum* and *G. hirsutum*, Oluoch et al. (2016), identified eight QTL regions contributing to salt tolerance and Zheng et al. (2016) identified multiple QTLs contributing toward drought tolerance. By applying RNA-Seq technology, Zhang F. et al. (2016) identified differentially expressed genes from *G. davidsonii*, a superior salt tolerant diploid cotton species. Similarly, Fan et al. (2015) detected 109 *WRKY* genes from transcriptome analyses in a salt-tolerant wild cotton species *G. aridum* to investigate the roles of these genes in cotton salt tolerance. From another salt-tolerant wild cotton species, *G. klotzschianum*, genes regulating salt tolerance mechanisms such as hormone biosynthesis and signal transduction, reactive oxygen species, and salt overly sensitive signal transduction related genes were elucidated though RNA-seq experiments from a time controlled NaCl (300 mM) experiments at 0, 3, 12, and 48 h intervals (Wei et al., 2017).

## Soybean

**Figure 4:**
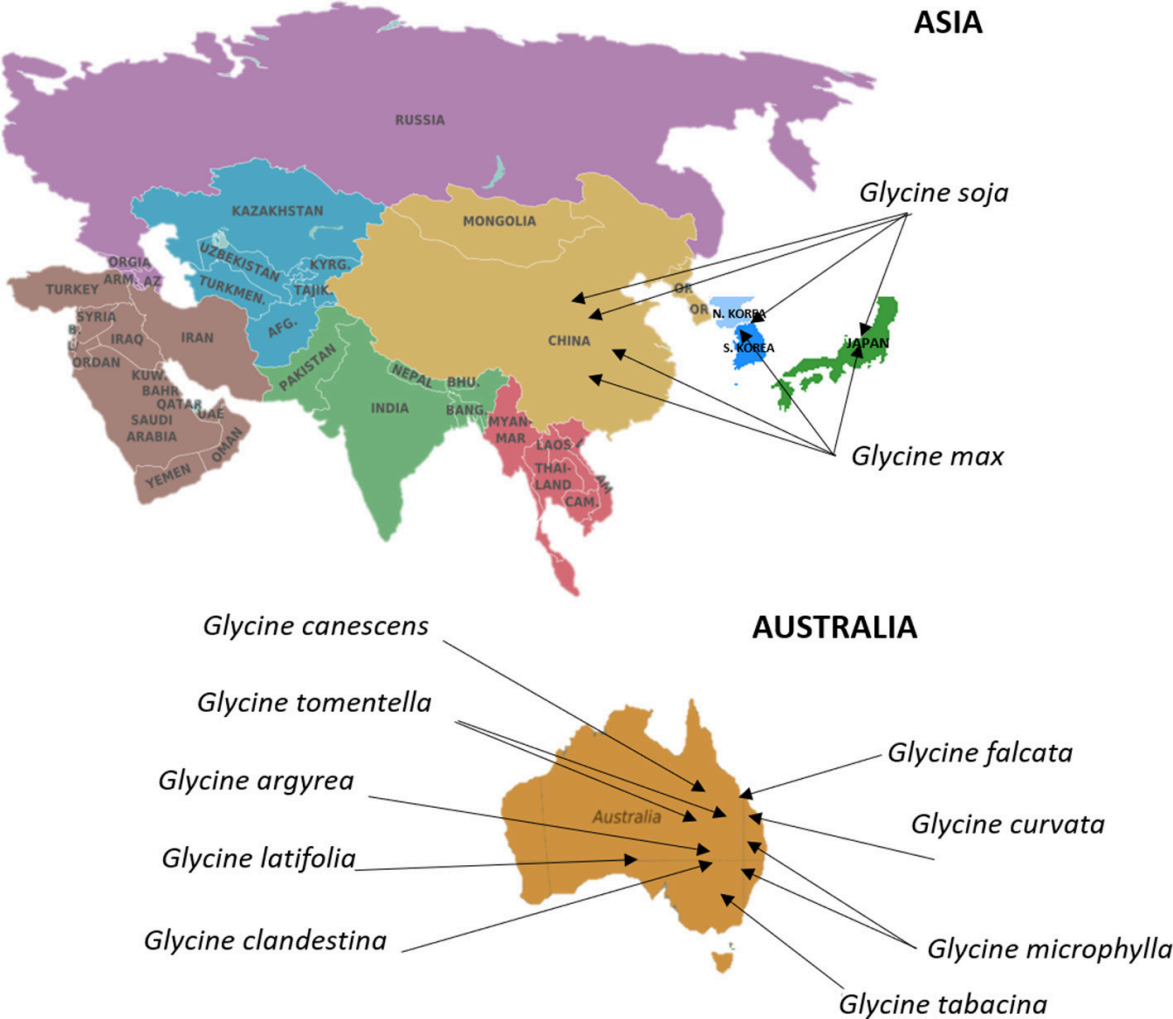
Centers of origin and primary geographical distribution of cultivated soybean and its wild relatives. Information on the distribution and centers of origin was gathered from Brown et al. (1989), Sherman-Broyles et al. (2014), Zhou et al. (2015). The Germplasm Resources Information Network in Beltsville, USA (https://npgsweb.ars-grin.gov/gringlobal/search.aspx) stores large global collection of soybean cultivars. Commonwealth Scientific and Industrial Research Organization in Canberra, Australia, encompasses a major collection of wild Glycine species.

Soybean (*Glycine max* [L.] Merr.) is an annual legume crop with major economic significance, contributing to more than half of the global oilseed production (Wilson, 2008). Domestication of cultivated soybean is thought to have happened in China ~5,000 years ago from wild soybean (*Glycine soja* Sieb. & Zucc.) (Boerma and Specht, 2004). Soybean introduction to the Korean peninsula and then Japan is estimated to have occured about 2,000 years ago, whereas, the crop first appeared in North America in 1765. Finally, soybean introduction to Central and South Americas happened during the first half of the last century (Wilson, 2008). Soybean has undergone natural phenotypic changes in plant development, flowering time, seed size and color, seed shattering, and dormancy during the process of domestication. Understanding the process of domestication and crop improvement at a genetic level is crucial for soybean breeding and crop improvement (Zhou et al., 2015). Genetic diversity of soybean has eroded due to domestication and breeding for yield to meet food demand. The genus *Glycine* consists of two subgenera, *Soja* and *Glycine*. Subgenus *Soja* includes *G. max* and its annual wild relative *G. soja. G. soja* has been distributed across broad geographical ranges and has a high genetic diversity due to the ability to adapt to a wide range of ecological conditions (Qiu et al., 2013; Sherman-Broyles et al., 2014). These geographical locations can be found for soybean in Figure 4. Because of the adaptability of *G. soja* it has become an important source of novel genes and alleles for soybean improvement necessary to meet the requirements of a rapidly growing world population and environmental changes (Table 4). The subgenus *Glycine* includes several perennial species such as *G. canescens, G. clandestina, G.latifolia, G. microphylla, G. tabacina, G. tomentella, G. falcate, G. argyrea, G. carvata, and G. hirticaulis*, which grow primarily in Australia (Brown et al., 1989; Sherman-Broyles et al., 2014; Zhou et al., 2015; Figure 4).

**Table 4:** Summary of biotic and abiotic stress tolerance traits of wild relatives of cultivated soybean (*Glycine max*).

**Traits of interest**	**Crop wild relative**	**Putative cause of resistance/tolerance**
**TOLERANCE TO BIOTIC STRESSES**		
**Nematode tolerance**		
Soybean Cyst Nematode resistance	*G. soja* *G. tomentella* *G. argyrea* *G. pescadrensis*	cqSCN-006 and cqSCN-007 Unknown Unknown Unknown
**Disease tolerance**		
Asian Soybean Rust resistance	*G. canescens* *G. clandestine* *G. tabacina* *G. tomentella* *G. argyrea* *G. latifolia* *G. microphylla*	Unknown Unknown Unknown Unknown Unknown Unknown Unknown
Sclerotina Stem Rot resistance	*G. tabacina* *G. tomentella*	Unknown Unknown
Powdery Mildew resistance	*G. canescens* *G. tomentella*	Unknown Unknown
**TOLERANCE TO ABIOTIC STRESSES**		
Drought tolerance	*G. soja*	Lower epidermal conductance, high relative water content (RWC), higher osmotic adjustment (OA), high level of water use efficiency (WUE)
	*G. latifolia*	Unknown
	*G. canescens*	Unknown
Salt tolerance	*G. soja*	Synthesis of compatible solutes, induction of reactive oxygen species (ROS), lower cell membrane permeability

### Tolerance to biotic stresses

#### Nematode tolerance

Soybean cyst nematode (SCN) (*Heterodera glycines*) is one of the major pathogens that affects soy. Concibido et al reviewed research efforts toward the identification of new sources of SCN-resistance both in cultivated soybean and *G. soja* as of 2004 (Concibido et al., 2004). Several new reports on the discovery of novel SCN-resistance QTL(s) in *G. soja* have been published based on traditional genetic linkage mapping or linkage disequilibrium based association mapping (Winter et al., 2007; Vuong et al., 2015; Zhang H. et al., 2016; Yu and Diers, 2017). Stacking of three SCN resistance alleles, *rhg1-a, rhg1-b*, and *Rhg4*, from the cultivated *G. max* with two SCN-resistance QTLs, cqSCN-006, and cqSCN-007, derived from *G. soja*, resulted in more durable resistance to the disease (Kim et al., 2011). Among 223 *G. tomentella* plant introductions tested, 86 accessions showed resistance to three *H. glycines (*HG) types, HG0, HG2, and HG1.2.3, 69 accessions showed resistance to two HG types and 22 showed resistance to one HG type. Of the other 12 perennial Glycine species evaluated, all accessions of *G. argyrea* and *G. pescadrensis* were resistant to all three HG types (Wen et al., 2017). So far no SCN resistance genes derived from soybean's CWR have been cloned.

#### Disease tolerance

Numerous diseases caused by bacteria, fungi, nematodes, oomycetes, and viruses have played a significant role in soybean production resulting in tremendous yield losses among susceptible varieties. Compared to the first report published in 1975 (Sinclair and Backman, 1993), the fifth edition of the *Compendium of Soybean Diseases and Pests* shows the dramatic increase in the number of diseases affecting soybean (Hartman et al., 2015) which is believed to have resulted from the accelerated growth in production and expansion of the crop to new geographies (Hartman et al., 2011). Molecular mechanisms of soybean disease resistance have been extensively studied. The majority of soybean disease resistance (*R*) genes cloned to date have encoded proteins with two conserved motifs, including nucleotide binding sites (NBS) and leucine rich repeats (LRR). NBS-LRR genes confer resistance to pathogens through a gene-for-gene interaction mechanism (Innes et al., 2008).

Asian soybean rust (ASR) is a major fungal disease caused by *Phakopsora pachyrhizi* and has the potential to cause significant soybean yield loss. Perennial *Glycine* species have demonstrated resistance to this pathogen. Screening of 294 accessions from 17 Glycine species identified ASR resistance sources within *G. canescens, G. clandestine, G. tabacina, G. argyrea, G. latifolia, G. microphylla*, and *G. tomentella* (Hartman et al., 1992). Other research studies toward the identifying new sources of ASR resistance have been reviewed by Langenbach et al. (2016).

Sclerotinia stem rot, commonly known as white mold, is caused by the pathogen *Sclerotinia sclerotiorum*. This pathogen is one of most important diseases in North and South America and ranked second to SCN yield reductions within the USA. Several accessions of *G. tabacina* and *G. tomentella* showed partial resistance to *S. sclerotiorum* (Hartman et al., 2000). SCN resistance loci were also identified in *G. lalifolia* (Chang, 2015). Several accessions of *G. canescens* and *G. tomentella* showed resistance to powdery mildew disease and sudden death syndrome (Mignucci and Chamberlain, 1978; Hartman et al., 2000).

### Tolerance to abiotic stresses

#### Drought tolerance

Genotypic variation for epidermal conductance, relative water content and osmotic adjustment was assessed in 58 *G. max* genotypes, *G. soja*, and nine genotypes from six different perennial wild soybean. Results suggested that accessions of *G. latifolia* and *G. canescens* are more likely to be drought tolerant than *G. max* and *G. soja* due to their lowered epidermal conductance, relatively higher water content, and higher osmotic adjustment (James et al., 2008). PI407155, a genotype of *G. soja*, demonstrated a higher level of water use efficiency compared to “Essex” genotype of *G. max* (Chen et al., 2006).

#### Salinity tolerance

CWRs of soybean have shown a notable level of tolerance to soil salinity. It has been reported that *G. max* and *G. soja* have reciprocal mechanisms of tolerance to soil salinity: *G. soja* accessions showed a higher leaf tolerance to Cl^−^ toxicity than *G. max* but were more susceptible to Na^+^ accumulation (Luo et al., 2005). Tolerance to salinity in *G. soja* was believed to be due to the release of sodium ions which subsequently reduced the accumulation at toxic concentrations in plant organs. At the biochemical level, it is believed that induction of plant hormones, reactive oxygen species, cell membrane modifications, and synthesis of compatible solutes control the observed salt tolerance of *G. soja* (Lu et al., 2008). A single dominant gene derived from the wild soybean accession PI483463 was reported to control salt tolerance (Lee et al., 2009). The gene *GsWRKY20*, which encodes a WRKY-type transcription factor, was isolated from *G. soja* and was capable of increasing salt and drought tolerance of susceptible alfalfa after transformation. Relatively lower membrane permeability and lower malondialdehyde content were also observed in the transgenic alfalfa, as well as, a higher accumulation of free proline and soluble sugars compared with wild-type plants under high-salinity and water-deficit conditions (Tang et al., 2014). Over-expression of G. soja gene, *GsJAZ2*, in Arabidopsis resulted in enhanced plant tolerance to salt and alkali stress (Zhu et al., 2012).

## Emerging technologies for the identification, characterization, and transfer of traits from CWRs

Although wild relatives of crops are an important source of genetic diversity, their genepool has not been sufficiently explored (Li Y. H. et al., 2014). These wild relatives can increase the adaptive capacity of agricultural systems around the world by offering new allelic variations that are required to address disease pressures, farming practices, market demands, and climatic conditions. However, the process of introducing genetic diversity from wild species into cultivars requires a significant amount of time, resources, and human capacity (Dempewolf et al., 2017). Molecular markers and genotyping platforms can enhance our ability to rapidly dissect useful agronomic traits found in wild relatives and transfer these traits to elite germplasm through QTL introgression, marker assisted selection, and other genomic approaches. The recent developments of 50 K maize, 50 K soybean, 50 K rice, and 63 K cotton SNP arrays will further aid in the detection of useful variation from wild relatives through QTL mapping, genome wide association studies, and genomic selection studies. These studies could also be used for positive selection of traits of interest and negative selection of wild genomic segments causing agronomic penalties in interspecific hybridization and pre-breeding programs (Ganal et al., 2011; Song et al., 2013; Hulse-Kemp et al., 2015; Singh et al., 2015).

Wide availability of cost-effective next generation sequencing technologies have created opportunities to generate whole genome sequence for several crop species and their wild relatives. These genome resources enhance our ability to mine economically important traits from wild relatives through structural, functional and comparative genomics approaches. Availability of reference genome sequences for maize (Schnable et al., 2009; Lai et al., 2010), rice (Matsumoto et al., 2005; Kawahara et al., 2013), soybean (Haun et al., 2011), cotton diploid progenitors (Paterson et al., 2012; Wang et al., 2012; Li F. et al., 2014), cultivated Upland cotton (Li et al., 2015; Zhang et al., 2015), and Pima cotton (Yuan et al., 2015) has further created opportunities to generate draft assemblies for wild relatives enhancing our ability to mine economically important traits in these gene pools using robust bioinformatics tools (Sripathi et al., 2016). The generation of genomic resources, such as those listed above, from the wild relatives of major crops would allow for characterization of their genome contents and structural variations to understand the evolution, as well as, enable large scale comparisons of wild relatives to their cultivated counterparts to realize the benefits of a Pan-genome for a crop species. The Pan-genome would constitute a common sets of genes available at the species level along with unique gene sets available within a subset of individuals, including wild relatives. Such meta-analyses would enable genome wide comparison of gene pools across the genetic stocks and enhance our understanding of evolutionary history of domesticated crop species, genetic erosion during the domestication process, and shed light on avenues for recovering useful genetic variation lost during domestication from these wild relatives. Pan-genome studies on wild relatives of maize have elucidated the underlying genetic basis of two traits important for fitness and adaptation: timing of the juvenile-to-adult vegetative and vegetative-to-reproductive developmental transitions (Hirsch et al., 2014). In soybean, Pan-genome studies have helped identify soybean lineage-specific copy number variants allowing for positive selection for biotic resistance, seed composition, flowering and maturity time, organ size, and final biomass (Li Y.H. et al., 2014). Exploitation of novel genes and alleles in wild relatives through pan-genome studies would prove highly valuable in cotton to overcome the diminishing genetic gains in yield, fiber quality, biotic, and abiotic stress tolerance caused by narrow gene pools in cultivated cotton species.

Genomic technologies are providing a holistic perspective of gene structure, organization, and regulation in the genome, as well as, their role in biological pathways; and such information greatly accelerates crop breeding (Huang et al., 2016). The functional roles of genes and alleles are being explored through transcription and translational genomic studies to identify genes impacting the crop productivity positively. Such genes have been candidates for testing the cross-transferability of phenotype to other crops and organisms through transformation experiments. In cotton, one of the most highly accepted transgenic crops across the globe, transformation has proved highly challenging. However, recent genome editing technologies and novel plant breeding techniques are promising to allow for manipulation of gene sequences in crops. Sequence-specific nucleases such as zinc finger nucleases (ZFNs), transcription activator-like effector nucleases (TALENs), or clustered, regularly interspaced short palindromic repeat–associated endonucleases (CRISPR/Cas) are typically introduced through transformation and create double stranded breaks at the target locus due to their sequence specificity and can also add or delete nucleotides at that site. Transgenes and other off-target mutations can be further removed through self- or back-crossing (Huang et al., 2016). Knowledge of the novel genes associated with valuable traits from wild relatives can be sourced to make changes through targeted mutagenesis in the genes of cultivated crops to introduce a new phenotype or variation to improve crop performance. Genome editing of bacterial leaf blight disease susceptible S genes in rice such as the SWEET14 promoter and SWEET-type S genes using TALENs (Li et al., 2012) and CRISPR-Cas9 (Zhou et al., 2014) systems have resulted in resistant genotypes. Van de Wiel et al. have thoroughly reviewed deployment of these genome editing technologies on candidate genes controlling disease resistance, product quality, allergens, male fertility, yield, and other traits (van de Wiel et al., 2017). In order to take advantage of genome editing tools for crop improvement, apart from the knowledge of large effect gene variants from wild relatives, development of highly efficient transformation systems is necessary.

## Conclusion

Diverse biotic and abiotic stresses, climate change, and rapidly increasing demand for food production are posing unprecedented challenges for global agriculture. Development of superior cultivars by harnessing diverse sources of variation and modern molecular and genomic tools have the potential to improve food production process. While plant domestication over the millennia has contributed to development of numerous cultivars in crop plants, those efforts primarily relied on yield improvement, edibility, and very few other traits. Such a strong selection has led to the creation of genetic bottlenecks and thereby resulted in the reduction of genetic variation. In contrast, CWRs in their natural environments have constantly been challenged and maintained higher levels of genetic diversity. Leveraging the untapped genetic diversity available in CWRs for improvement of crops is an attractive option for improving crops. The use of modern technologies for identifying and dissecting the molecular, genetic, and genomic bases of traits in CWRs can accelerate this process. The review presented here has discussed the wealth of traits in CWRs of four important crops and the efforts that have gone in this area of research toward harnessing this valuable resource for crop improvement.

## Author contributions

JM, SK, SG, and RB have substantially contributed to the conception, design, and writing of this review paper. KP has contributed to the critical review of entire article and improved the quality and uniformity of the language. IA has contributed to the expansion and critical review of cotton section.

### Conflict of interest statement

JM, SG, RB, KP, and SK were employed by company Dow AgroSciences. The remaining author declares that the research was conducted in the absence of any commercial or financial relationships that could be construed as a potential conflict of interest.
